# Subjective Xerostomia and Chairside Salivary Dysfunction in Institutionalized Older Adults: Concordance and Exploratory Derivation of a Five-Item Short Form

**DOI:** 10.3390/dj14090557

**Published:** 2026-09-02

**Authors:** Mădălina Monica Bicheru, Andreea Zamfirescu, Elena Preoteasa, Adrian Iustin Georgevici, Nicolae Vladimir Bicheru, Cristina Teodora Preoteasa

**Affiliations:** 1 Department of Prosthodontics, Faculty of Dentistry, “Carol Davila” University of Medicine and Pharmacy, 020021 Bucharest, Romania; 2 Department of Geriatrics and Gerontology, Faculty of Midwifery and Nursing, “Carol Davila” University of Medicine and Pharmacy, 050474 Bucharest, Romania; 3 Katholisches Klinikum, Ruhr-University Bochum, 44801 Bochum, Germany; 4 Department of Scientific Research Methods-Ergonomics, Faculty of Dentistry, “Carol Davila” University of Medicine and Pharmacy, 020021 Bucharest, Romania

**Keywords:** xerostomia, salivary dysfunction, stimulated salivary output, chairside salivary dysfunction, patient-reported outcome measures, geriatric dentistry, institutionalized older adults

## Abstract

**Background/Objectives**: In institutionalized older adults, we examined the concordance between self-reported xerostomia and chairside salivary dysfunction and explored whether a smaller subset of Xerostomia Inventory items could retain comparable screening performance. **Methods**: This cross-sectional study included institutionalized older adults. The 11-item Xerostomia Inventory was compared with a six-marker chairside whole-saliva panel, with all variables scaled 0 (normal) to 1 (pathological). We measured how closely symptoms tracked each objective marker, how well the questionnaire identified hyposalivation, and whether fewer questions could be comparable with the full 11-item inventory. **Results**: The study included 90 participants, with a mean age of 79.5 years; 55.6% were women. Symptoms tracked every marker in the expected direction (correlation τ-b 0.32 to 0.62). The questionnaire identified objective hyposalivation with an area under the ROC curve of 0.87 (95% CI 0.78–0.96) and substantial chance-corrected agreement (Cohen’s κ 0.69, 95% CI 0.51–0.87). A five-item short form reproduced the objective findings as well as the full questionnaire did (cross-validated R^2^ 0.56 ± 0.03 versus 0.56). **Conclusions**: In this high-prevalence institutional cohort, self-reported symptoms agreed with graded salivary function more closely than the older discordance literature would suggest, though still within the range of recent studies rather than beyond it. A five-item subset, chosen for its alignment with the objective salivary axis rather than for internal consistency, recovered as much as the full inventory, which points to a short, function-oriented score as a plausible first-stage screen. We would treat this short form as a candidate first-stage screening approach that requires external validation, rather than as a replacement for objective salivary assessment or as a validated replacement for the Xerostomia Inventory.

## 1. Introduction

Older adults living in long-term residential care constitute a clinically and socially vulnerable population, frequently characterized by frailty, multimorbidity, dependence on caregivers, limited financial resources, and restricted access to oral healthcare [1,2]. In this setting, salivary dysfunction should not be regarded simply as a consequence of chronological aging, but rather as the cumulative expression of systemic disease and treatment exposure [3]. Chronic conditions requiring multiple medications [4], particularly agents with anticholinergic or other xerogenic effects, may reduce salivary secretion and impair oral lubrication, thereby increasing the likelihood of xerostomia, hyposalivation, swallowing difficulties, and related oral complications [5,6].

Dry mouth may refer either to xerostomia, the patient’s subjective feeling of oral dryness, or to hyposalivation, a measurable reduction in salivary flow. Although these conditions often occur together, they do not always overlap. Some patients report marked dryness despite having normal salivary flow, whereas others show reduced salivary secretion without experiencing noticeable symptoms [7,8,9]. Although objective salivary testing remains the gold standard to confirm glandular hypofunction, symptom questionnaires are useful for identifying perceived discomfort.

However, the relationship between perceived oral dryness and measured salivary flow is not consistent across all settings. Studies using dry-mouth questionnaires have reported widely varying levels of agreement with objective salivary measurements, depending on the population examined, the scoring method, and the definition of salivary hypofunction [10,11,12]. Much of the earlier evidence was obtained from outpatient populations, which may not reflect the situation of institutionalized older adults. In this group, dry mouth is particularly common and is often linked to polypharmacy and exposure to medications with anticholinergic effects [6,13,14]. As a result, reduced salivary function is frequently present. This raises the question of whether a smaller selection of Xerostomia Inventory items could reflect objective salivary measurements as accurately as the full questionnaire [10].

The primary objective of this study was to assess how closely the total score and individual items of the 11-item Xerostomia Inventory reflected objective salivary function in institutionalized older adults. The secondary objective was to evaluate how well the questionnaire identified and measured hyposalivation and to determine which symptoms were most strongly associated with reduced salivary function. As an exploratory objective, we examined whether a shorter, objectively anchored subset of items could achieve screening performance comparable to that of the full questionnaire.

## 2. Materials and Methods

### 2.1. Study Design and Participants

A cross-sectional study was carried out among older adults living in social care centers for dependent persons in Romania. Recruitment and clinical assessments took place between September 2025 and February 2026. During the study period, all residents who fulfilled the eligibility criteria were invited to participate. Eligible residents were aged 60 years or older, had complete edentulism in at least one jaw, and were able to understand the study questions and cooperate with the clinical and salivary assessments. Residents with communication or comprehension difficulties that prevented reliable questionnaire completion or participation in the examination were excluded.

Ninety residents were enrolled and included in the final analysis. The sample size was determined by the number of eligible residents available in the participating institutions during the recruitment period who agreed to participate. Accordingly, the study used a convenience sample rather than a probability-based sampling strategy. No a priori sample-size calculation was performed because the study was exploratory and recruitment was constrained by the size of the accessible institutional population. The findings, particularly those related to short-form derivation, should therefore be interpreted as exploratory and hypothesis-generating and require confirmation in an independent cohort.

The study protocol was approved by the Ethics Committee of the “Carol Davila” University of Medicine and Pharmacy, Bucharest, Romania (approval No. 22332/5 September 2025). All procedures were performed in accordance with the Declaration of Helsinki. Written informed consent was obtained from every participant before study procedures began.

The study was reported in accordance with the Strengthening the Reporting of Observational Studies in Epidemiology recommendations for cross-sectional studies. The completed STROBE checklist is provided as Supplementary Table S1.

### 2.2. Data Collection and Examiner Roles

Information was collected through a structured interview, administration of the Xerostomia Inventory, chairside assessment of salivary function, and review of the residents’ institutional medical records. Medical history and medication information were checked against the available records whenever possible. These clinical data were collected to characterize the broader health context of the participants but were not included as covariates in the present analyses. The analytical focus of this study was the concordance between subjective xerostomia measures and chairside salivary function and the exploratory derivation of an objectively anchored subset of Xerostomia Inventory items, rather than the identification of independent clinical or pharmacological determinants of salivary dysfunction.

To limit the possibility that knowledge of the subjective responses could influence interpretation of the salivary findings, the two components of the assessment were performed by different examiners. One examiner administered the Xerostomia Inventory and recorded the participants’ answers. A second examiner carried out the salivary tests and graded the visual and colorimetric findings according to predefined criteria. The questionnaire and salivary results were recorded separately. The same assessment procedures and scoring rules were applied to all participants.

### 2.3. Subjective Assessment of Oral Dryness

Subjective oral dryness was assessed with the 11-item Xerostomia Inventory (XI), a validated questionnaire that examines the frequency and functional impact of dry-mouth symptoms [15]. The XI was administered as a structured interview rather than as a self-completed form. This approach allowed the examiner to present each question consistently and facilitated participation among older residents who might have had visual, literacy, or physical limitations. Each item was scored on a five-point Likert scale ranging from 1, “never”, to 5, “very often”. Higher scores represented more frequent dry-mouth symptoms.

For the statistical analyses, the score for each item was transformed to a scale from 0 to 1. A value of 0 represented the lowest symptom frequency, whereas a value of 1 represented the highest frequency. The XI total score was calculated as the mean of the 11 transformed item scores. Consequently, higher XI values indicated a greater subjective burden of oral dryness. The total XI score and the individual item scores were both retained for analysis. The total score represented overall symptom burden, while the item-level analysis was used to determine which specific complaints were most closely associated with impaired salivary function.

### 2.4. Objective Assessment of Salivary Function

Objective salivary function was evaluated using a six-parameter chairside panel comprising unstimulated whole-saliva flow, mucosal hydration, resting saliva viscosity, resting salivary pH, stimulated salivary output, and buffering capacity. Unstimulated whole saliva was collected at rest over a 5 min period into a graduated collection container, following established procedures for whole-saliva collection. The collected volume was recorded after 5 min and used to classify unstimulated salivary output according to the predefined criteria [16]. The other five parameters were examined using the GC Saliva-Check BUFFER kit (GC Corporation, Tokyo, Japan), following the manufacturer’s instructions and established chairside whole-saliva assessment procedures [3,16,17]. The kit provides clinical and semi-quantitative categories of salivary function. The resulting grades were therefore treated as ordered chairside assessments and not as continuous laboratory measurements. The chairside assessment and standardized severity coding of each marker are summarized in Table 1.

For the binary screening analysis, objective hyposalivation was defined as reduced stimulated output, that is, a GC grade of 2 (low) or 1 (very low). This is an operational, semi-quantitative endpoint and should not be read as equivalent to laboratory-confirmed hyposalivation based on a continuous gravimetric flow-rate threshold. Accordingly, the chairside classifications used in this study should be regarded as pragmatic indicators of salivary dysfunction rather than substitutes for standardized quantitative sialometry.

### 2.5. Standardization of Subjective and Objective Variables

All variables, subjective and objective, were placed on a common 0–1 severity scale (0 = normal or least affected, 1 = most pathological), with the coding for each marker shown in Table 1. Because low GC grades denote poorer function, the GC grades were reversed so that higher standardized values consistently indicate greater impairment.

This transformation preserved the order and clinical severity of the original categories without converting the chairside grades into continuous physiological measurements. On this scale, a positive subjective–objective association denotes concordance, that is, greater perceived dryness accompanying more severe measured impairment.

### 2.6. Statistical Analysis

First, associations between subjective xerostomia and objective salivary function were examined using Kendall’s rank correlation coefficient (τ-b), which was chosen because the salivary markers were ordinal and the transformed XI item scores retained their ordered structure. Kendall’s τ-b was calculated between each XI item, as well as the XI total score, and each of the six objective salivary markers. To account for multiple testing across the item-by-marker comparisons, *p*-values were adjusted using the Benjamini–Hochberg procedure.

Second, we investigated how well the questionnaire total separates affected from unaffected residents using the area under the receiver operating characteristic (ROC) curve, which uses the full range of scores and does not depend on any chosen cut-off. For clinical readability, we also report sensitivity and specificity at the cut-off that performed best in this sample, together with Cohen’s κ for chance-corrected agreement. Because more than three-quarters of the residents were affected, κ is held down by that imbalance, so we add two coefficients that are less sensitive to it: the first-order agreement coefficient of Gwet (AC1) and the prevalence-adjusted bias-adjusted κ (PABAK) [18,19].

Third, we examined whether a smaller subset of XI items could retain the objective information captured by the full inventory. The six chairside salivary markers were summarized by their first principal component, which was used as the dominant objective salivary-severity axis. All possible combinations of one to six XI items were evaluated, and for each subset size the combination showing the highest alignment (R^2^) with the objective axis was identified. Item selection was performed within a nested cross-validation procedure, so that subset selection occurred only in the training data and performance was evaluated in the held-out residents [20,21]. The final subset size was chosen from the parsimony curve by considering the smallest number of items that recovered essentially the same objective-axis information as the full XI. This approach represents criterion-based item selection, in which items are selected according to their association with an external objective criterion rather than according to internal consistency alone [22].

## 3. Results

### 3.1. Sample and Bivariate Concordance

The cohort and the prevalence of objective pathology are summarized in Table 2.

**Figure 1:**
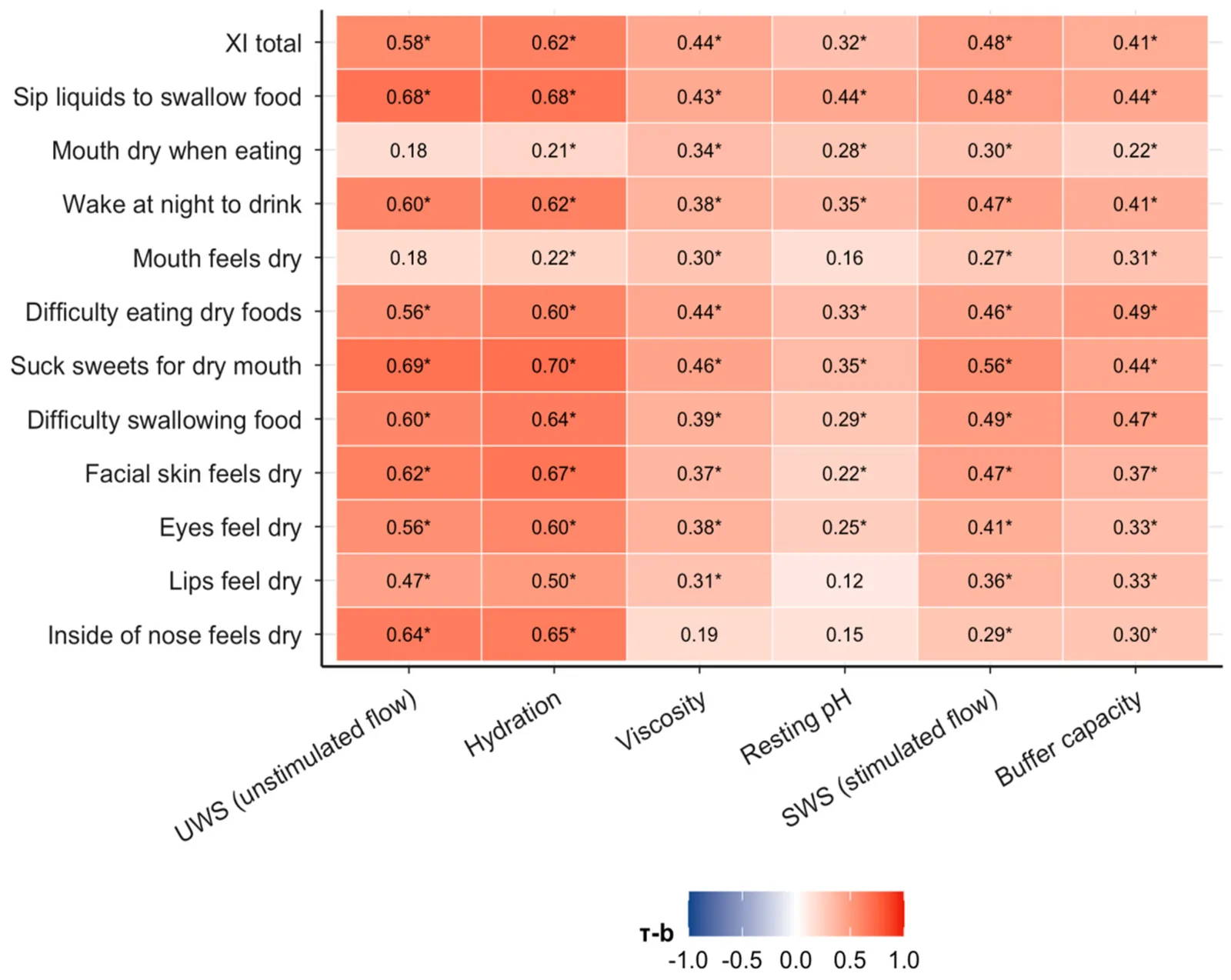
Kendall rank correlation (τ-b) of each Xerostomia Inventory item (and the total) with each objective salivary marker. Rows are subjective items (0 = no symptom to 1 = maximum), columns are objective markers (0 = normal to 1 = pathological); positive values (red) denote concordance. An asterisk marks cells significant after Benjamini–Hochberg adjustment across the full item-by-marker family.

Residents who reported more dry-mouth symptoms had more abnormal chairside findings on every one of the six markers (Figure 1). Of the 72 item-by-marker correlations, 66 remained significant after adjustment for multiple testing. For the questionnaire total, the association was closest with mucosal hydration (τ-b 0.62) and with unstimulated flow (0.58), and weakest with resting pH (0.32): symptoms tracked how moist the mouth was more closely than they tracked its acidity.

### 3.2. Screening for Objective Hyposalivation

**Figure 2:**
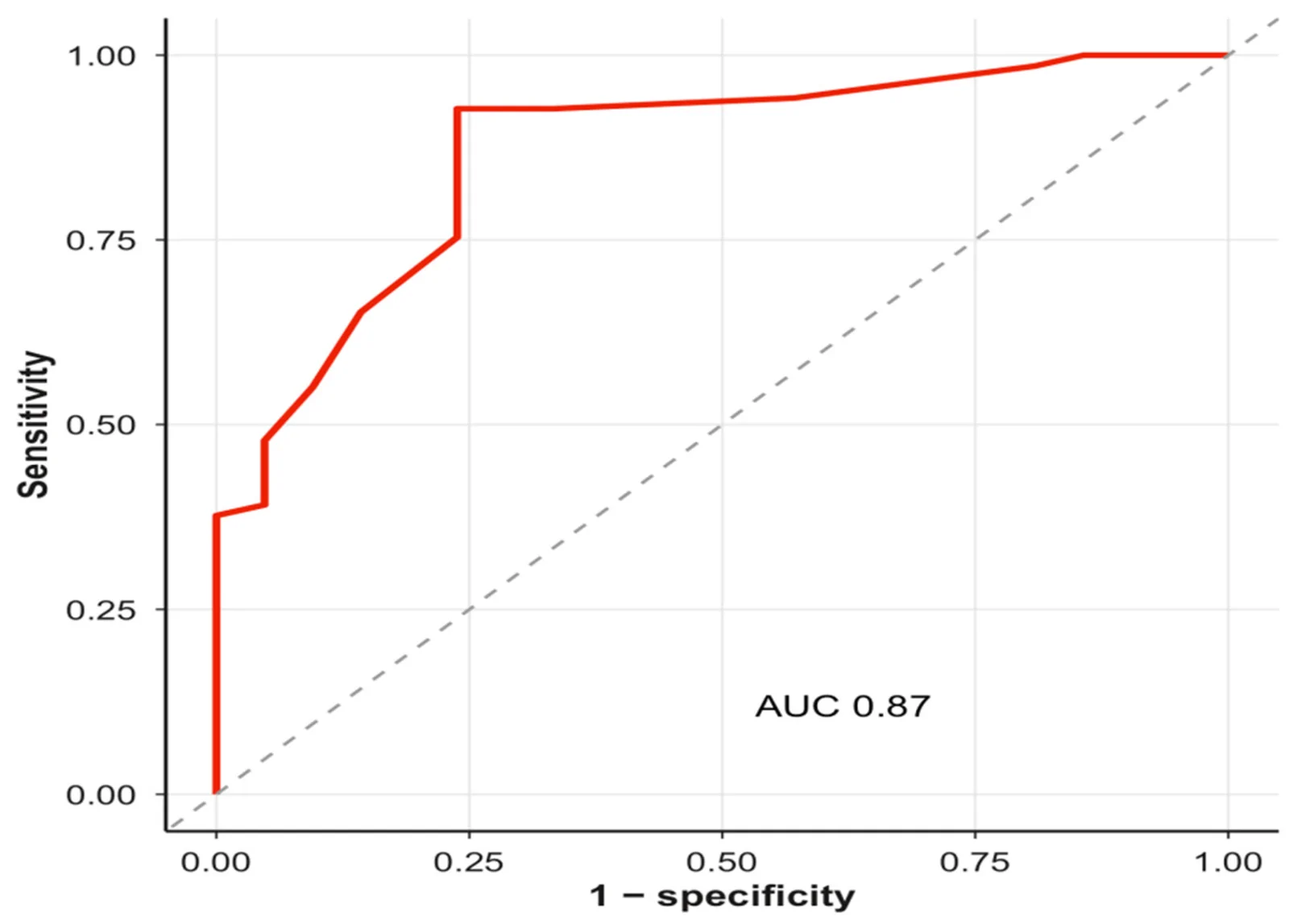
Receiver operating characteristic curve of the Xerostomia Inventory total for objective hyposalivation (low stimulated whole-saliva flow). The dashed diagonal marks chance; the area under the curve, annotated on the plot, is threshold-free and so does not depend on the operating point.

Reduced stimulated flow, the objective definition of hyposalivation used here, was present in 69 of the 90 residents (76.7%). Against that endpoint, the questionnaire total discriminated well, with an AUC of 0.87 (Table 3, Figure 2). In practical terms, if one affected and one unaffected resident are picked at random, the affected resident has the higher symptom score about 87 times in 100. At the cut-off that performed best in this sample, sensitivity was 92.8% and specificity 76.2%. Chance-corrected agreement was substantial, at a κ of 0.69. Because more than three-quarters of the residents were affected, that κ is held down by the imbalance itself [18], while the prevalence-robust coefficients sit higher, at an AC1 of 0.83 and a PABAK of 0.78 (Table 3). These adjusted coefficients should be read as complementary to κ rather than as independent corroboration of it, because where the condition is this common, AC1 exceeds κ almost by construction [19,23]; the agreement figures are therefore in part a property of the high objective base rate and may not carry over to healthier populations. At the item level, the strongest screening signals were observed for functional or compensatory complaints, particularly “suck sweets for dry mouth”, “difficulty swallowing food”, and “sip liquids to swallow food”.

### 3.3. An Objective-Anchored Short Form

**Figure 3:**
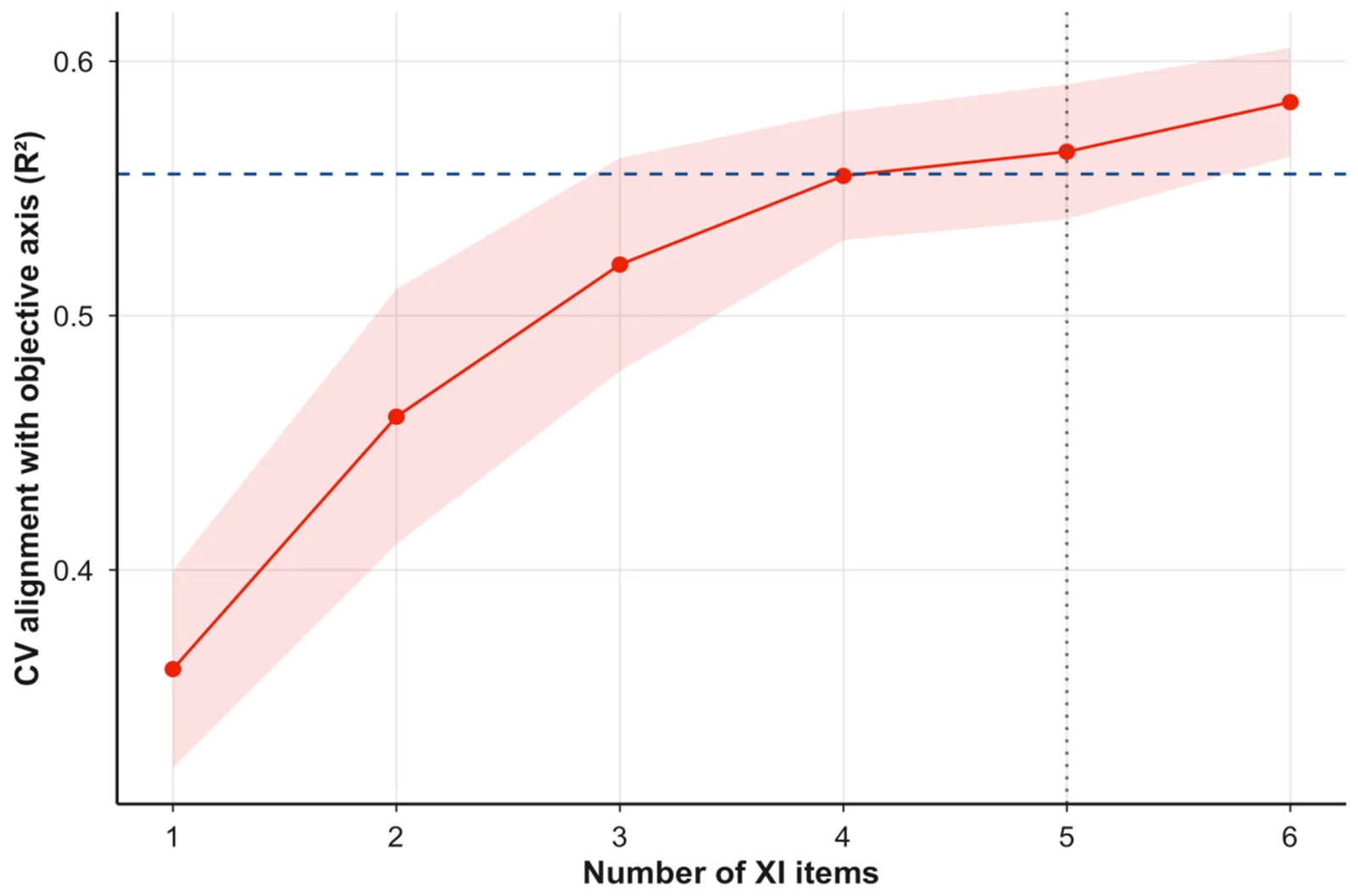
Objective-anchored parsimony curve: nested cross-validated alignment (R^2^ with the dominant objective axis) versus number of Xerostomia Inventory items, from exhaustive best-subset selection. The shaded band is ±1 standard error across folds. The dashed horizontal line marks the full 11-item inventory; the dotted vertical line marks the chosen short-form size.

The six chairside markers largely move together: a single underlying dimension of salivary severity accounts for 72.9% of their combined variation. We therefore investigated how many XI items were needed to recover this objective salivary-severity dimension while keeping the number of questions as small as possible. As shown by the parsimony curve in Figure 3, performance increased as additional items were included and approached that of the full XI at five items. The candidate five-item subset was therefore selected as a parsimonious solution that retained the objective salivary-severity information while reducing the number of questionnaire items. Moreover, the selection procedure evaluated combinations of items under nested cross-validation rather than simply ranking individual items according to their R^2^ or AUC values [22].

The resulting candidate objective-anchored subset comprised five items: “sip liquids to swallow food”, “lips feel dry”, “suck sweets for dry mouth”, “mouth feels dry”, and “mouth dry when eating”. At the score level, the objective-anchored five-item subset showed a cross-validated R^2^ of 0.56 with the objective salivary-severity axis, the same numerical value as the full 11-item XI. The established, psychometrically derived five-item Summated Xerostomia Inventory (SXI-5) reproduced somewhat less of the same objective dimension (R^2^ = 0.49). Because only the objective-anchored subset was derived using this objective criterion, this difference is descriptive and does not establish superiority or formal non-inferiority [23,24]. For reduced stimulated salivary output, the objective-anchored five-item subset showed a cross-validated AUC of 0.90 (apparent AUC = 0.93), compared with 0.87 for the full XI. This numerical difference was not formally tested and should therefore not be interpreted as evidence that the five-item subset performs better than the full inventory.

As shown in Table 4, the objective-anchored subset was not simply composed of the five items with the highest individual R^2^ values. The strongest individual associations with the objective salivary-severity axis were observed for “suck sweets for dry mouth” (R^2^ = 0.46), “sip liquids to swallow food” (R^2^ = 0.45), “difficulty swallowing food” (R^2^ = 0.41), “difficulty eating dry foods” (R^2^ = 0.41), and “facial skin feels dry” (R^2^ = 0.34). Only two of these items were retained in the candidate subset, whereas “difficulty swallowing food”, “difficulty eating dry foods”, and “facial skin feels dry” were not. This difference reflects the multivariable selection strategy, which evaluated the information provided jointly by combinations of items under nested cross-validation rather than selecting items solely according to their individual performance [22].

For comparison with an established short-form instrument, the candidate objective-anchored subset and the SXI-5 shared three items: “lips feel dry”, “mouth feels dry”, and “mouth dry when eating”. The candidate subset additionally included “sip liquids to swallow food” and “suck sweets for dry mouth”, whereas the established SXI-5 includes “difficulty eating dry foods” and “difficulty swallowing food” [23,24]. Thus, the two five-item forms share a core of three direct oral-dryness symptoms but differ in the two additional items retained.

## 4. Discussion

In this cross-sectional cohort of 90 institutionalized older adults, self-reported dry-mouth symptoms were consistently associated with objectively assessed salivary impairment. The XI total score showed positive associations with all six salivary markers, and the questionnaire discriminated reduced stimulated salivary output according to the GC Saliva-Check BUFFER classification with an AUC of 0.87. At the item level, functional or compensatory complaints, particularly sucking sweets to relieve dryness, difficulty swallowing food, and sipping liquids to facilitate swallowing, showed some of the strongest associations with objective salivary dysfunction.

The objective-anchored five-item subset retained the same cross-validated association with the dominant salivary axis as the complete 11-item XI. Its discrimination of reduced stimulated salivary output was also comparable with that of the full questionnaire. These findings support the further evaluation of a brief symptom-based screening approach, but they do not establish formal non-inferiority, because no prespecified non-inferiority margin or independent validation cohort was used. The five-item score should therefore be regarded as a candidate short form rather than as an already validated replacement for the XI [20,21,22,23,24]. Importantly, the proposed short form is not intended to replace objective assessment of salivary function. If its performance is confirmed by external validation, its potential role would be as a first-stage screening instrument to identify individuals who may benefit from subsequent clinical evaluation and standardized objective salivary testing.

Xerostomia and hyposalivation remain clinically distinct. Patients may report substantial oral dryness despite apparently normal salivary secretion, whereas objectively reduced flow may occur in the absence of perceived dryness. Nevertheless, recent studies show that the magnitude of this discordance varies considerably according to the population, the questionnaire used, the scoring method, and the objective salivary endpoint. Reported discrimination has ranged from approximately 0.59 to 0.90, placing the AUC of 0.87 observed in our cohort toward the upper end of, but still within, the recent published range [7,8,9,10,11,12].

The item-level findings also agree with recent evidence that the total XI score may conceal clinically relevant differences between symptoms. In the study reporting relatively weak total-score discrimination, item-based models performed better, and “mouth feels dry” and “lips feel dry”—both retained in our short form—were among the complaints most closely related to measured flow. Recent work in adults exposed to anticholinergic medication has similarly shown that reduced unstimulated salivary flow contributes independently to XI severity. In another medication-induced xerostomia cohort, objectively reduced salivary secretion and greater anticholinergic exposure were associated with higher XI scores, while eating- and drinking-related behaviors reflected the functional consequences of oral dryness. These observations support the prominence of oral, swallowing, and compensatory items in our analyses [10,25,26,27].

The present results should not, however, be interpreted as evidence that strong subjective–objective agreement is universal among institutionalized older adults. A recent study of dependent residents found only a weak association between the SXI-5 and unstimulated salivary flow, although objective clinical signs of dryness were related to flow. Our study also differs methodologically because the objective panel consisted mainly of ordinal chairside categories rather than continuously measured gravimetric flow rates. The use of separate examiners for questionnaire administration and salivary assessment reduced the risk that knowledge of the subjective responses directly influenced the objective grading, but it does not remove the measurement limitations inherent to semi-quantitative testing [12].

The importance of the objective reference is further illustrated by research in Sjögren’s syndrome. Błochowiak found that reported xerostomia was associated with unstimulated whole-saliva flow but not with focal lymphocytic sialadenitis, focus score, or serological and inflammatory markers. Although Sjögren’s syndrome represents a specific disease context, the comparison is included here only to illustrate that subjective symptoms may correspond differently to functional, histological, or systemic measures. The present study did not classify xerostomia according to etiology, and disease-specific comparisons should therefore be interpreted cautiously [28].

Several characteristics of the present cohort may explain the relatively close relationship observed. Reduced stimulated output was present in more than three quarters of residents, while normal viscosity, resting pH, and buffering capacity were uncommon. The AUC answers a different question: how well the score separates affected from unaffected residents, although it too can shift with the range of severity present in a sample [18,19,23,28,29].

The item-level results provide some insight into why the objective-anchored five-item subset performed similarly to the full XI. Three direct dryness symptoms—“lips feel dry”, “mouth feels dry”, and “mouth dry when eating”—were shared by the candidate subset and the established SXI-5, suggesting that the direct perception of oral dryness represents an important core component of both short forms. However, these direct dryness items were not consistently the strongest individual correlates of the objective salivary measures. As shown in Figure 1 and Table 4, functional or compensatory symptoms, including “sip liquids to swallow food”, “difficulty swallowing food”, “difficulty eating dry foods”, and “suck sweets for dry mouth”, also showed relatively strong associations with objective salivary dysfunction. This may be clinically meaningful because direct dryness complaints primarily capture the perception of oral dryness, whereas eating- and swallowing-related complaints may reflect the functional consequences of insufficient oral lubrication, and behaviors such as sipping liquids or sucking sweets may reflect attempts to compensate for these symptoms [28]. Thus, combining direct dryness perception with functional or compensatory information may capture complementary aspects of salivary dysfunction that are not fully represented by direct dryness symptoms alone. This provides a possible explanation for why the addition of functional or compensatory items allowed the five-item subset to retain a similar proportion of the objective salivary-severity signal as the full XI. This interpretation is exploratory and should be confirmed in an independent cohort.

Mastication and swallowing depend jointly on dentate and prosthodontic status, salivary flow, and food consistency [30]. Because all participants had complete edentulism in at least one jaw and detailed prosthodontic status was not incorporated into the present concordance models, some of the associations involving eating- and swallowing-related XI items may also reflect prosthodontic or masticatory factors [31]. This should be considered when interpreting the relatively strong associations between these functional complaints and measured salivary function.

This functional emphasis is consistent with, but not identical to, the established SXI-5. Three of the five objectively anchored items overlap with the psychometrically derived short form: “lips feel dry”, “mouth feels dry”, and “mouth dry when eating”. The present score additionally retained two compensatory behaviors that aligned more closely with the objective salivary panel. This difference reflects the purpose of item selection: the SXI-5 was designed to preserve the psychometric content of the original inventory, whereas the present subset was selected against an external physiological criterion. The omitted extra-oral items remained individually associated with some salivary markers; their exclusion indicates redundancy after the functional items were included, not a complete absence of clinical information [6,22,23,24].

We used reduced stimulated salivary output according to the GC Saliva-Check BUFFER classification as the primary binary objective endpoint. This was a more conservative outcome in the present data than the unstimulated chairside classification, for which the XI showed stronger discrimination. However, the stimulated outcome remains an ordinal chairside classification and should not be presented as equivalent to laboratory-confirmed hyposalivation based on a continuous gravimetric threshold. Standardized sialometry remains the objective reference when precise physiological quantification is required [16,30]. Consequently, the diagnostic performance reported here refers specifically to the chairside classification used in this study and should not be assumed to represent performance against standardized gravimetric sialometry.

The principal methodological contribution is not the introduction of a new questionnaire or salivary test, but the alignment of item selection with an external objective salivary criterion. The first principal component accounted for 72.9% of the variance in the objective panel, suggesting that the measurements largely reflected a common salivary-severity dimension. The five-item mean recovered the same proportion of this objective axis as the complete XI under cross-validation. This provides a transparent explanation of which symptoms carried most of the objective information and why removing the remaining items produced little loss in performance [20,21,22,32].

The clinical relevance of screening salivary dysfunction in residential care extends beyond the symptom of dryness itself. In a recent study of 589 institutionalized older adults, hyposalivation occurred within a broader pattern involving frailty, prosthesis use, candidiasis, and subprosthetic stomatitis. These associations do not establish causality, but they show that reduced salivary function may identify residents with wider oral and functional vulnerability [33].

The potential usefulness of a brief screen is particularly relevant in Romania. Cosoroabă et al. described limited access to preventive dental services among institutionalized older adults, together with financial, logistical, and organizational barriers. In such settings, a short symptom-based score could help prioritize residents for medication review, oral examination, and confirmatory salivary testing [2,4]. The intended role of such a symptom-based score would therefore be limited to first-stage triage: it could help identify residents who should undergo further clinical assessment, but it cannot establish salivary gland hypofunction and should not replace objective salivary testing.

Because the five questions do not require dental equipment, they could potentially be administered by a trained nurse, caregiver, or other non-dental healthcare professional. However, this mode of administration was not tested. Its feasibility, inter-rater reliability, training requirements, referral threshold, diagnostic performance, and cost-effectiveness must be assessed prospectively before routine implementation. The present findings therefore support a possible first-stage triage pathway, not an established non-dental diagnostic program.

## 5. Limitations

Several limitations bound the interpretation of these findings. Most residents were impaired on most objective markers, and the small numbers of normal viscosity, pH, and buffering-capacity observations limit the precision of marker-specific comparisons. The high and uneven prevalence influenced the agreement statistics and reduced their transportability to healthier populations. Unstimulated flow and mucosal hydration were almost interchangeable in this cohort and were not included together in multivariable models. The sample of 90 also limits complex interaction modeling and may contribute to optimistic performance estimates, despite the use of nested cross-validation [11,12,18,19,23,29]. Unstimulated flow was classified as reduced in 68 of the 90 residents in the present dataset, whereas the companion report of this cohort records 69 [34]. The difference concerns a single resident and affects none of the statistics reported in either paper, all of which are anchored on the Xerostomia Inventory total and on stimulated whole-saliva flow.

Medication burden, systemic diseases, and prosthodontic status were not included as covariates in the present analyses. These factors are clinically relevant determinants of xerostomia and salivary dysfunction and may influence both subjective symptom reporting and objective salivary findings. Their exclusion reflects the focused analytical aim of the present study rather than an assumption that they are unimportant. Given the sample size and the exploratory short-form derivation, expanding the models to include multiple clinical and pharmacological covariates would also have increased model complexity relative to the available number of participants. Future studies in larger independent cohorts should examine whether the observed subjective–objective associations remain robust after accounting for medication exposure, comorbidity, frailty, and prosthodontic characteristics.

The chairside salivary measurements were ordinal or categorical rather than laboratory-grade continuous sialometry. Although the subjective and objective components were assessed by separate examiners, visual and colorimetric interpretation remains susceptible to measurement variability. The convenience sample included only residents able to provide consent and cooperate with the procedures and may therefore underrepresent individuals with more severe cognitive, communicative, or functional impairment. All participants were recruited from a restricted Romanian institutional setting and had complete edentulism in at least one jaw, further limiting generalizability [33,34,35].

Finally, the study was cross-sectional, and all observed relationships represent concordance or association rather than causation or temporal prediction. The candidate short form, its scoring system, and any future referral threshold require external validation in an independent cohort using blinded, standardized sialometry and a wider spectrum of salivary function, including a pre-specified head-to-head comparison against alternative item subsets, such as one composed of the individually strongest items. Future studies should also examine administration by non-dental staff, test–retest reliability, feasibility, and the clinical consequences of false-positive and false-negative classifications.

## 6. Conclusions

In institutionalized older adults, self-reported xerostomia was consistently associated with chairside indicators of salivary dysfunction, and the full Xerostomia Inventory discriminated reduced stimulated salivary output with good accuracy. An objective-anchored five-item subset retained a cross-validated association with the dominant salivary dysfunction axis comparable to that of the full inventory. This subset may represent a candidate first-stage screening approach for identifying residents who warrant further assessment; however, it is not a substitute for objective salivary testing. External validation in an independent and adequately powered cohort, preferably using standardized continuous sialometry as the objective reference, is required before any clinical implementation.

## Figures and Tables

**Table 1:** Chairside assessment and standardized severity coding of the six-marker whole-saliva panel. Five markers were graded with the GC Saliva-Check BUFFER kit against its reference charts; unstimulated flow was assessed separately. Grades were treated as ordered chairside categories, not continuous laboratory values, and GC grades were reversed so that higher standardized values denote greater impairment.

Marker	Chairside Assessment	Categories	Coding (0–1)
Unstimulated whole-saliva flow	Whole saliva collected at rest for 5 min into a graduated container	Normal/reduced	0/1
Mucosal hydration	Everted, blotted lower lip; time for minor-gland droplets to reappear (GC scale)	Normal/impaired	0/1
Resting saliva viscosity	Visual inspection of resting saliva against GC chart	Watery-clear/frothy/sticky-viscous	0/0.5/1
Resting salivary pH	Colorimetric strip on resting saliva (GC scale)	Normal/moderately/severely abnormal	0/0.5/1
Stimulated salivary output	Volume after paraffin chewing (GC three-level scale)	Normal (3)/low (2)/very low (1)	0/0.5/1
Buffering capacity	Three-pad colorimetric strip on stimulated saliva (GC chart)	Normal/low/very low	0/0.5/1

**Table 2:** Sample characteristics for the full resident cohort. Age is mean (SD); the Xerostomia Inventory total is median [IQR] on its 0 to 1 scale. Each objective salivary marker is summarized as the number (percentage) of residents in any pathological grade, with markers scored 0 = normal to 1 = pathological.

Characteristic	Value
N	90
Female	50 (55.6%)
Age, mean (SD)	79.5 ± 7.1
XI total, median [IQR]	0.50 [0.41, 0.61]
Pathological: UWS (unstimulated flow)	68 (75.6%)
Pathological: Hydration	69 (76.7%)
Pathological: Viscosity	82 (91.1%)
Pathological: Resting pH	84 (93.3%)
Pathological: SWS (stimulated flow)	69 (76.7%)
Pathological: Buffer capacity	86 (95.6%)

**Table 3:** Screening performance and chance-corrected agreement of the Xerostomia Inventory total for objective hyposalivation (low stimulated whole-saliva flow). The prevalence carries a Wilson interval; the area under the ROC curve a DeLong interval; sensitivity, specificity, and predictive values a Wilson interval; and Cohen’s κ a normal-approximation interval. Sensitivity, specificity, and predictive values are taken at the in-sample Youden cut and are therefore optimistic. At this cut false positives equal false negatives, so the positive and negative predictive values coincide with sensitivity and specificity. Gwet AC1 and PABAK are prevalence-robust agreement coefficients reported alongside κ.

Measure	Value
Hyposalivation prevalence	69/90 (76.7%, 95% CI 66.9–84.2%)
AUC (95% CI)	0.87 (0.78–0.96)
Sensitivity	92.8% (84.1–96.9%)
Specificity	76.2% (54.9–89.4%)
PPV	92.8% (84.1–96.9%)
NPV	76.2% (54.9–89.4%)
Cohen’s κ (95% CI)	0.69 (0.51–0.87)
Gwet AC1	0.83
PABAK	0.78

**Table 4:** Item-level comparison between the candidate objective-anchored five-item subset derived in the present study and the established five-item Summated Xerostomia Inventory (SXI-5). R^2^ represents the individual association of each XI item with the objective salivary-severity axis; AUC represents the individual discrimination of reduced stimulated salivary output; τ-b flow represents Kendall’s rank correlation with stimulated salivary flow. Check marks indicate inclusion in the candidate objective-anchored subset derived in the present study and/or in the established SXI-5.

Item	Complaint	Objective-Anchored Subset	Established SXI-5	R^2^	AUC	τ-b Flow
XI-6	suck sweets for dry mouth	✓		0.46	0.86	0.56
XI-1	sip liquids to swallow food	✓		0.45	0.87	0.48
XI-7	difficulty swallowing food		✓	0.41	0.86	0.49
XI-5	difficulty eating dry foods		✓	0.41	0.79	0.46
XI-8	facial skin feels dry			0.34	0.82	0.47
XI-3	wake at night to drink			0.32	0.81	0.47
XI-9	eyes feel dry			0.26	0.80	0.41
XI-10	lips feel dry	✓	✓	0.19	0.74	0.36
XI-11	inside of nose feels dry			0.18	0.75	0.29
XI-4	mouth feels dry	✓	✓	0.13	0.69	0.27
XI-2	mouth dry when eating	✓	✓	0.11	0.69	0.30

## Data Availability

The dataset analyzed during the current study is available from the corresponding authors upon reasonable request, in accordance with privacy and ethical restrictions. Analytic code is maintained in a private project repository and can be shared upon request for academic review.

## Supplementary Materials

The following supporting information can be downloaded at: https://www.mdpi.com/article/10.3390/dj14090557/s1. Table S1: The completed STROBE checklist for cross-sectional studies.
