# Supplementary material for: Subjective Xerostomia and Chairside Salivary Dysfunction in Institutionalized Older Adults: Concordance and Exploratory Derivation of a Five-Item Short Form
Source: Dent J (Basel). 2026 Sep 2;14(9):557. doi: 10.3390/dj14090557 (PMC13605887; doi:10.3390/dj14090557)
Supplement: Supplementary file 1 [file dentistry-14-00557-s001.zip › dentistry-4484606-supplementary.pdf]

SUPPLEMENTARY TABLE S1

STROBE Checklist for Cross-Sectional Studies

Manuscript Title: Subjective Xerostomia and Chairside Salivary Dysfunction in Institutionalized Older Adults: Concordance and Exploratory Derivation of a Five-Item Short Form

This study was reported in accordance with the STROBE statement for cross-sectional studies.

Item	Description	Location
1a Title	Cross-sectional study of subjective xerostomia and chairside salivary dysfunction, with exploratory derivation of a five-item short form, in institutionalized older adults	Title
1b Abstract	Structured abstract (Objectives, Methods, Results, Conclusions) with associational framing	Abstract
2 Background	Distinction between xerostomia and hyposalivation; variable subjective-objective concordance across settings; rationale for a shorter, objectively anchored screening subset	Introduction (Section 1)
3 Objectives	Primary: concordance of the Xerostomia Inventory total and items with objective salivary function. Secondary: screening for measured hyposalivation. Exploratory: objective-anchored short form	Introduction (Section 1)
4 Study design	Cross-sectional	Methods (Section 2.1)
5 Setting	Social care centres for dependent persons, Romania; recruitment and assessment September 2025-February 2026	Methods (Section 2.1)
6 Participants	Age >=60 years, complete edentulism in at least one jaw, able to complete questionnaire and examination; residents with communication or comprehension difficulties excluded; written informed consent	Methods (Section 2.1); Results (Section 3.1)
7 Variables	11-item Xerostomia Inventory (subjective); six-marker chairside whole-saliva panel: unstimulated flow, mucosal hydration, viscosity, resting pH, stimulated output, buffering capacity (objective)	Methods (Sections 2.3, 2.4); Table 1
8 Data sources/measurement	Structured interview for the Xerostomia Inventory; GC Saliva-Check BUFFER kit and separate unstimulated-flow assessment; institutional medical records	Methods (Sections 2.2, 2.3, 2.4); Table 1
9 Bias	Questionnaire and salivary assessments performed by two different examiners, recorded separately; semi-quantitative chairside grading; convenience sample restricted to residents able to consent and cooperate	Methods (Section 2.2); Limitations (Section 5)

Item	Description	Location
10 Study size	Ninety residents; convenience sample determined by eligible residents available; no formal a priori sample-size calculation (exploratory study)	Methods (Section 2.1); Limitations (Section 5)
11 Quantitative variables	All subjective and objective variables placed on a common 0-1 severity scale; GC grades reversed so higher values denote greater impairment; objective hyposalivation defined as reduced stimulated output (GC grade 2 or 1)	Methods (Sections 2.4, 2.5); Table 1
12a Statistical methods (primary)	Kendall rank correlations, Mann-Whitney comparisons and Jonckheere-Terpstra trend tests for concordance; ROC/AUC, Youden-cut sensitivity and specificity, and chance-corrected agreement (Cohen kappa, Gwet AC1, PABAK) for screening	Methods (Section 2.6)
12b Statistical methods (subgroups)	Objective-anchored short form selected by exhaustive best-subset search against the dominant objective axis under nested cross-validation; Benjamini-Hochberg adjustment within families; unstimulated flow excluded from multivariable models due to near-duplication with hydration	Methods (Section 2.6)
13 Participants	Ninety residents enrolled and analysed;	Methods (Section 2.1); Results (Section 3.1)
14 Descriptive data	Age, sex, Xerostomia Inventory total, and prevalence of pathological grades for each objective marker	Results (Section 3.1); Table 2
15 Outcome data	Prevalence of objective hyposalivation (reduced stimulated flow) and distribution of symptom scores	Results (Sections 3.1, 3.2); Tables 2 and 3
16 Main results	Item-by-marker concordance with Benjamini-Hochberg adjustment; AUC 0.87 (95% CI 0.78-0.96) and Cohen kappa 0.69 for objective hyposalivation; five-item short form matching the full inventory on the objective axis	Results (Sections 3.1-3.3); Tables 3 and 4; Figures 1-3
17 Other analyses	Item-level associations; parsimony curve across subset sizes; comparison with the psychometric SXI-5 and the best single item; prevalence-robust agreement coefficients	Results (Sections 3.2, 3.3); Table 4; Figures 1-3
18 Key results	Self-reported symptoms consistently associated with chairside salivary impairment; functional and compensatory complaints carried the screening signal; five-item subset retained the objective association	Discussion (Section 4)
19 Limitations	Cross-sectional design; ordinal chairside measurements;	Limitations (Section 5)
20 Interpretation	Concordance and association rather than causation; the five-item score is a candidate short form requiring external validation, not a validated replacement for the Xerostomia Inventory	Discussion (Section 4); Conclusions (Section 6)
21 Generalisability	Restricted Romanian institutional setting; all participants edentulous in at least one jaw; limited applicability to healthier populations	Limitations (Section 5)
22 Funding	Listed in the main manuscript	Funding and declarations
